# Community structure affects trophic ontogeny in a predatory fish

**DOI:** 10.1002/ece3.2600

**Published:** 2016-12-20

**Authors:** Javier Sánchez‐Hernández, Antti P. Eloranta, Anders G. Finstad, Per‐Arne Amundsen

**Affiliations:** ^1^ Department of Zoology, Genetics and Physical AnthropologyUniversity of Santiago de CompostelaSantiago de CompostelaSpain; ^2^ Department of Arctic and Marine BiologyUiT The Arctic University of NorwayTromsøNorway; ^3^ Department of Natural HistoryNTNU University MuseumTrondheimNorway; ^4^ Aquatic Ecology DepartmentNorwegian Institute for Nature Research (NINA)TrondheimNorway; ^5^ Department of Biological and Environmental SciencesUniversity of JyväskyläJyväskyläFinland

**Keywords:** dietary switch, fish assemblage, individual specialization, interindividual variation, niche shift, predation

## Abstract

While most studies have focused on the timing and nature of ontogenetic niche shifts, information is scarce about the effects of community structure on trophic ontogeny of top predators. We investigated how community structure affects ontogenetic niche shifts (i.e., relationships between body length, trophic position, and individual dietary specialization) of a predatory fish, brown trout (*Salmo trutta*). We used stable isotope and stomach content analyses to test how functional characteristics of lake fish community compositions (competition and prey availability) modulate niche shifts in terms of (i) piscivorous behavior, (ii) trophic position, and (iii) individual dietary specialization. Northern Scandinavian freshwater fish communities were used as a study system, including nine subarctic lakes with contrasting fish community configurations: (i) trout‐only systems, (ii) two‐species systems (brown trout and Arctic charr [*Salvelinus alpinus*] coexisting), and (iii) three‐species systems (brown trout, Arctic charr, and three‐spined sticklebacks [*Gasterosteus aculeatus*] coexisting). We expected that the presence of profitable small prey (stickleback) and mixed competitor–prey fish species (charr) supports early piscivory and high individual dietary specialization among trout in multispecies communities, whereas minor ontogenetic shifts were expected in trout‐only systems. From logistic regression models, the presence of a suitable prey fish species (stickleback) emerged as the principal variable determining the size at ontogenetic niche shifts. Generalized additive mixed models indicated that fish community structure shaped ontogenetic niche shifts in trout, with the strongest positive relationships between body length, trophic position, and individual dietary specialization being observed in three‐species communities. Our findings revealed that the presence of a small‐sized prey fish species (stickleback) rather than a mixed competitor–prey fish species (charr) was an important factor affecting the ontogenetic niche‐shift processes of trout. The study demonstrates that community structure may modulate the ontogenetic diet trajectories of and individual niche specialization within a top predator.

## 1 Introduction

Ontogenetic changes in ecological processes are widespread across the animal kingdom, and they are central to understand ecosystem functioning (Nakazawa, 2015; Woodward et al., 2005). For example, ontogenetic trajectories can alter the structure of communities and ecosystem processes, such as respiration and net primary productivity (Rudolf & Rasmussen, 2013). In fact, studies of the mechanisms promoting trophic ontogeny are vital to improve our knowledge about how ecological communities are structured (van Leeuwen, Huss, Gårdmark, & De Roos, 2014; Takimoto, 2003). The timing and extent of ontogenetic niche shifts usually vary among individuals (see, e.g., Post, 2003), and they are determined by a suite of biotic and abiotic environmental conditions (e.g., Galarowicz, Adams, & Wahl, 2006; Kimirei et al., 2013; Olson, 1996). These include competitive interactions between sympatric species (i.e., species occupying the same geographical area), prey availability, predation risk, and internal mechanisms such as metabolic rate (Galarowicz et al., 2006; Kimirei et al., 2013; Olson, 1996; Sherwood, Pazzia, Moeser, Hontela, & Rasmussen, 2002; Werner, 1986). Ontogenetic dietary shifts have high ecological significance for organisms, as minimizing the ratio of mortality to growth over ontogeny may maximize lifetime reproductive output (Post, 2003; Werner, 1986; Wollrab, de Roos, & Diehl, 2013). In addition, ontogenetic dietary shifts can promote coexistence among sympatric species (Schellekens, De Roos, & Persson, 2010; Wollrab et al., 2013) and persistence of consumer‐resource dynamics (Takimoto, 2003), but also alter the energy flow and structure of food webs (Sánchez‐Hernández, 2016). Individual differences in ontogenetic dietary shifts can lead to development of ecologically distinct subpopulations, which, in turn, may cause different effects on community‐ and ecosystem‐level processes (De Roos & Persson, 2002; Olson, 1996). Moreover, interspecific interactions often play a key role in structuring communities, and these relationships are commonly influenced by ontogenetic niche shifts (Werner, 1986; Werner & Gilliam, 1984).

Elucidating the mechanisms behind ontogenetic niche shifts requires a framework that includes the complex interplay between community structure and individual diet variation. Generalist species can adapt their diets in response to spatial and temporal variation in prey availability and interspecific interactions (e.g., Beckerman, Petchey, & Morin, 2010). Thus, local community structure can largely determine ontogenetic dietary shifts by top predators, and this may have large influence on the structure and function of entire ecosystems (e.g., Bolnick et al., 2011; Werner, 1986; Woodward et al., 2005). Moreover, there has been a growing interest in the mechanisms promoting patterns of individual dietary specialization (e.g., Araújo, Bolnick, & Layman, 2011; Bolnick et al., 2003; Oudman et al., 2016), and recent studies in trophic ecology have focused on how individual diet variation may vary through ontogeny (Salvidio, Oneto, Ottonello, Costa, & Romano, 2015; Svanbäck, Quevedo, Olsson, & Eklöv, 2015; Zhao, Villeger, Lek, & Cucherousset, 2014). Overall, individuals usually become specialists through ontogeny by reducing their plasticity with age (Zhao et al., 2014; but see Vögler, Milessi, & Quiñones, 2003). The ontogenetic dietary shifts usually trigger changes in trophic position (Woodward & Hildrew, 2002), and in many cases, the trophic position increases with an animal's body size (e.g., Romanuk, Hayward, & Hutchings, 2011; Woodward & Hildrew, 2001), but not always (Layman, Winemiller, Arrington, & Jepsen, 2005; Schriever & Williams, 2013). However, few studies have investigated relationships between individual dietary specialization and trophic position through species' ontogeny. The recent study by Svanbäck et al. (2015) demonstrated that individual diet variation usually is highest at intermediate trophic positions and size classes. However, it has remained unexplored how local community structure shapes the relationships between ontogenetic niche shifts and individual diet variation.

Due to their relatively low productivity and simple food webs, the subarctic lakes in northern Scandinavia provide an excellent model system to study ontogenetic dietary shifts by top predators as a response to fish community structure (see, e.g., Hortal et al., 2014 for advantages of using lakes as model systems). These lakes are highly isolated and have simple fish communities consisting mainly of brown trout (*Salmo trutta* L.; henceforth trout), Arctic charr (*Salvelinus alpinus* (L.); henceforth charr), and/or three‐spined stickleback (*Gasterosteus aculeatus* L.; henceforth stickleback). Trout are generally the top predator and undergo ontogenetic dietary shifts from aquatic invertebrates to fish prey (Jensen, Kiljunen, & Amundsen, 2012; Klemetsen et al., 2003). Cannibalism occurs in some trout populations (e.g., L'Abée‐Lund, Langeland, & Sægrov, 1992), but the transition toward piscivory is often promoted by the presence of intermediate consumers, that is, suitable prey fish for top predators such as stickleback, charr, European minnow *Phoxinus phoxinus* (L.), or European whitefish *Coregonus lavaretus* (L.) (e.g., Jensen et al., 2012; L'Abée‐Lund, Aass, & Sægrov, 2002; Sánchez‐Hernández & Amundsen, 2015). Although interspecific interactions may change through ontogeny (e.g., Hin, Schellekens, De Roos, & Persson, 2011), charr is both a potential prey and a competitor species for trout (henceforth termed “mixed competitor–prey fish species”). The presence of both species in the same lake typically involves mixed competition–predation interactions that change through ontogeny (Persson et al., 2013): Large trout is usually able to feed on small charr, but the species generally compete for the same food resources when they are of the same size. Stickleback is a small‐sized fish species at intermediate trophic levels (henceforth termed “prey fish species”), often playing a central role in energy flow pathways to the lake top predators (Amundsen et al., 2009). The presence of charr and stickleback likely affects ontogenetic changes in trout feeding, considering the fundamental importance of prey availability and interspecific resource competition to diet and trophic position of top predators (Bolnick et al., 2011; Galarowicz et al., 2006; Woodward & Hildrew, 2002). In particular, the presence of sympatric fish species likely facilitates early piscivory as well as increased trophic position and degree of individual specialization among trout. Hence, the local fish community structure may influence the timing, degree, and prevalence of piscivory and the trophic position of trout in subarctic lakes (Jensen et al., 2012 and references therein). However, to the best of our knowledge, no previous studies have investigated how the local fish community structure shapes individual dietary specialization through trout ontogeny.

In the present study, we investigated how community structure, via its potential effects on prey availability and interspecific resource competition, affects ontogenetic dietary shifts of trout. We quantified ontogenetic dietary shifts of trout using three different measures: (i) prevalence of piscivory (based on presence/absence of prey fish in the stomach contents), (ii) individual dietary specialization [based on stomach content analyses (SCA)], and (iii) trophic position [based on stable isotope analyses (SIA)]. First, we investigated the general patterns of ontogenetic dietary shifts of trout in line with the above‐mentioned measures and thereafter the influence of community structure. Three different model fish community types with different levels of competition and prey availability were studied: (i) trout‐only systems, (ii) two‐species systems (trout coexisting with charr), and (iii) three‐species systems (trout coexisting with both stickleback and charr). We hypothesized that community structure—via competitive interactions with charr and predation on both stickleback and charr—influences ontogenetic niche shifts by trout. Specifically, we expected trout to show minor ontogenetic shift in trophic position in trout‐only systems, but a clear ontogenetic shift to a higher trophic position when sympatric fish species are present. This ontogenetic shift to a higher trophic position is expected to occur at a smaller size and be stronger (a steeper positive relationship between size and trophic position) in systems where both profitable small prey (stickleback) and mixed competitor–prey fish species (charr) are present, as compared to systems with only trout and charr. Finally, we hypothesized that community structure shapes the relationships between trophic position and individual dietary specialization of the top predator. In particular, we expected that the presence of profitable prey fish (stickleback), in conjunction with strong resource competition with charr, would induce higher individual dietary specialization by increasing piscivorous specialization by trout in multispecies communities as compared to systems with only trout and charr. The study provides novel insights into how community structure shapes individual dietary specialization through species' ontogeny.

## 2 Materials and methods

### 2.1 Sampling

**Figure 1:**
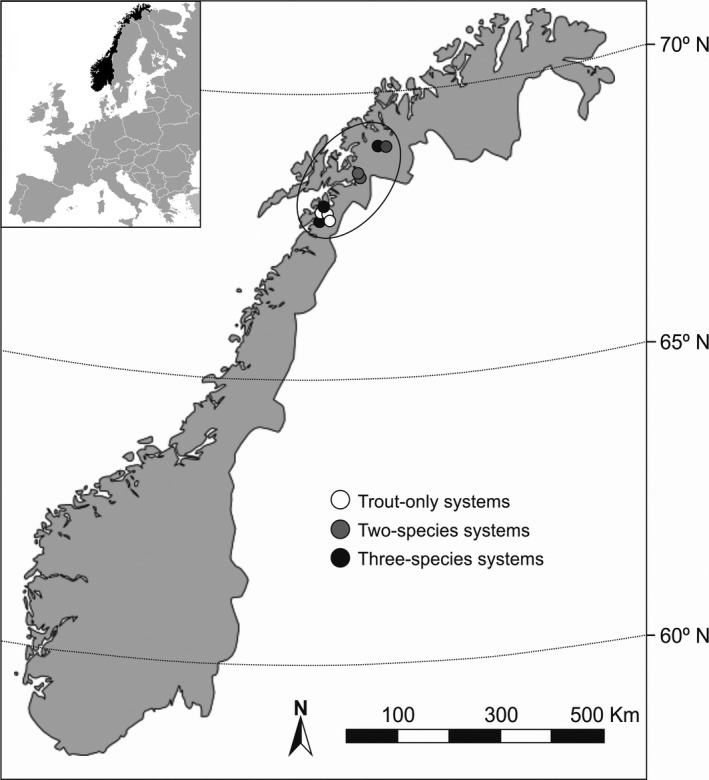
Location of the nine lakes in the study region (black ellipse), northern Norway

The present study was carried out in nine oligotrophic subarctic lakes located in northern Norway (Figure 1). The studied lakes included natural populations without fish introductions over the last decades. Sticklebacks were generally smaller than charr and thus more readily ingested by the trout, but juvenile charr of fork length below 100 mm were also suitable prey for large trout (see Table S1 for further size details).

The samples were collected in August 2010 (Fjellfrøsvatn and Takvatn), August 2013 (Forsanvatn, Makkvatn, Skilvatn, and Storvatn), and August 2014 (Jernvatnet, Sirkelvatn, and Slunkajavri). Fish were sampled in the littoral, profundal, and pelagic habitats using multimesh survey gillnets set overnight for 11–13 hr for 1–3 nights in each lake (see Eloranta, Knudsen & Amundsen 2013 and Sánchez‐Hernández & Amundsen, 2015 for details). Catch per unit effort (CPUE; the number of fish caught per 100 m^2^ gillnet per night) was estimated for each fish species from the littoral, pelagic, and profundal habitats using data from multimesh survey gillnet catches (Table S2). In all study lakes, trout were predominantly caught in the littoral zone but were also present in the pelagic zone in trout‐only and three‐species systems. Sticklebacks were only found in the littoral zone and charr were caught in all habitat types in the three‐species systems, whereas no pelagic individuals were caught in two of the two‐species systems (i.e., Jernvatnet and Sirkelvatn; Table S2).

The fish were measured (fork length, mm), dissected and the stomachs were removed and preserved in 96% ethanol for later diet analysis in the laboratory. A small sample of dorsal muscle tissue was dissected and stored at −20°C for later stable isotope analyses (SIA). Zooplankton were collected for SIA by taking several hauls throughout the pelagic water column with a 50‐ to 100‐μm mesh plankton net until sufficient material was obtained and prepared as described in Eloranta et al. (2015). Macrozoobenthos were collected from the littoral zone using a kick net in shallow water and an Ekman grab or a benthic sledge with a 500‐μm mesh in deeper littoral areas. Macrozoobenthos samples were separated from unwanted material (sediment, detritus, and vegetation) and sorted to the lowest feasible taxonomic group (mostly genus or family). The whole body of both benthic and pelagic invertebrates was stored at −20°C for later SIA, except molluscs and case‐making caddisflies, for which only the soft body tissue was used.

### 2.2 Stomach content analyses

In the laboratory, the stomachs were opened and the percentage of total fullness was visually determined, ranging from empty (0%) to full (100%) (see subjective methods in Hyslop, 1980). Prey items were identified to the lowest taxonomic level possible, and their contributions to the total stomach fullness were estimated according to Amundsen, Gabler, and Staldvik (1996). Detailed results from the SCA are provided as Supplementary material (Fig. S1).

To study the individual dietary specialization, the proportional similarity (PS_i_) index (Bolnick, Yang, Fordyce, Davis, & Svanbäck, 2002) was calculated using the lowest feasible taxonomic level standardized across the study lakes. This index compares each individual's diet to that of the entire population, with values ranging between 0 and 1. For individuals specializing on a single or few prey types, the PS_i_ values tend to be low, whereas for individuals that consume resources in a similar proportion as the entire population, the PS_i_ values approach 1 (Bolnick et al., 2002). Here, individual dietary specialization was expressed as the inverse of PS_i_ values (1 − PS_i_), meaning that individual dietary specialization is high when inverse values approach 1.

### 2.3 Stable isotope analyses

Samples from fish dorsal muscle, macrozoobenthos, and zooplankton were dried (48 hr in a freeze‐drier or at 60°C in an oven), ground to a fine powder, and precisely weighed (0.5–0.6 mg) for subsequent SIA. Stable carbon and nitrogen isotope ratios (expressed as δ^13^C and δ^15^N, respectively) were analyzed by an elemental analyzer coupled to a continuous flow isotope ratio mass spectrometer. The samples collected in 2010 (Fjellfrøsvatn and Takvatn) and 2013 (Forsanvatn, Makkvatn, Skilvatn, and Storvatn) were analyzed with a FlashEA 1112 elemental analyzer connected to a Thermo Finnigan DELTA Plus Advantage Mass Spectrometer at the University of Jyväskylä, Finland. In contrast, the samples collected in 2014 (Jernvatnet, Sirkelvatn and Slunkajavri) were analyzed at the Environmental Isotope Laboratory, University of Waterloo, on a Delta Plus Continuous Flow Stable Isotope Ratio Mass Spectrometer (ThermoFinnigan, Bremen, Germany) coupled to a 4010 Elemental Analyzer (Costech International S. p. A., Milan, Italy). In both laboratories, Vienna PeeDee Belemnite and atmospheric nitrogen were used as international references for carbon and nitrogen, respectively. Standard deviation of an internal working standard was <0.3 ‰ for δ^13^C and 0.2 ‰ for δ^15^N. Finally, stable carbon and nitrogen isotope ratios were used to estimate the trophic position (TP) according to two‐source isotopic mixing model of Karlsson and Byström (2005).

### 2.4 Statistics

Statistical analyses and graphical outputs were performed using R 3.2.2 (R Core Team 2015). The data showed non‐normality indicated by quantile–quantile (QQ) plots (Fig. S2) and Shapiro–Wilk tests (Table S3). Nonparametric Kruskal–Wallis test (three samples) was used to analyze differences in TP and 1 − PS_i_ among fish community types (trout‐only, two‐species and three‐species systems). Logistic regression models were fitted using the “popbio” package in R (Stubben & Milligan, 2007) to explore the probability of ontogenetic shift to piscivory of trout as a function of trout fork length based on presence/absence data (1 = fish prey found and 0 = no fish prey found in the trout stomach). The logistic regression models were fitted separately for each fish community type (using pooled data from all lakes having the same fish community). The shift to piscivory is assumed to take place when the probability of finding fish prey in the diet is 50% according to the fitted curve for the logistic regression model (Kahilainen & Lehtonen, 2003). Hence, this 50% probability level was employed to test whether the shift to piscivory is influenced by the fish community structure. Additionally, the strength of association between fish abundance (CPUE) and piscivory (%, prevalence) was tested using Pearson's rank correlation. This analysis allowed us to test how environmental gradients (here fish abundance) might explain the patterns in piscivory of trout.

Because our data did not meet normality and hence the assumptions for linear regression models (Zuur, Ieno, Walker, Saveliev, & Smith, 2009), we used generalized additive mixed models (GAMMs) to test whether fish community structure affects the ontogenetic shifts in trophic position and individual dietary specialization of trout, as well as the relationships between trophic position and individual dietary specialization. GAMMs were fitted using the package “mgcv” (Wood, 2015) employing cubic regression splines separately for each fish community type and using lake as a random factor. In this analysis, TP and 1 − PS_i_ were the dependent variables and length was the smoothed variable. When 1 − PS_i_ was modeled over TP, 1 − PS_i_ was the dependent variable and TP was the smoothed variable. An important term in GAMMs is the number of knots (*k*), which determines the smoothness of the curve; the more knots used, the less smooth the curve becomes (Zuur et al., 2009). Three knots are recommended if there are <30 observations and five knots if there are more than 100 observations (Keele, 2008), but increasing the number of knots increases the risk of overfitting (Zuur et al., 2009). The smoothness selection can be chosen based on either Akaike information criteria (AIC) or a visual comparison of the smoothers (Zuur et al., 2009). We chose the “optimal” model in terms of amount of knots based on visual comparison of the smoothers, including three knots to prevent overfitting and to adjust our data into realistic models. Finally, we ran sensitivity analyses in order to test whether the patterns of GAMMs remain the same after excluding large individuals (>400 mm fork length), which were not captured in trout‐only systems (Table S1). A significance level of *p *=* *.05 was used in all analyses.

## 3 Results

**Figure 2:**
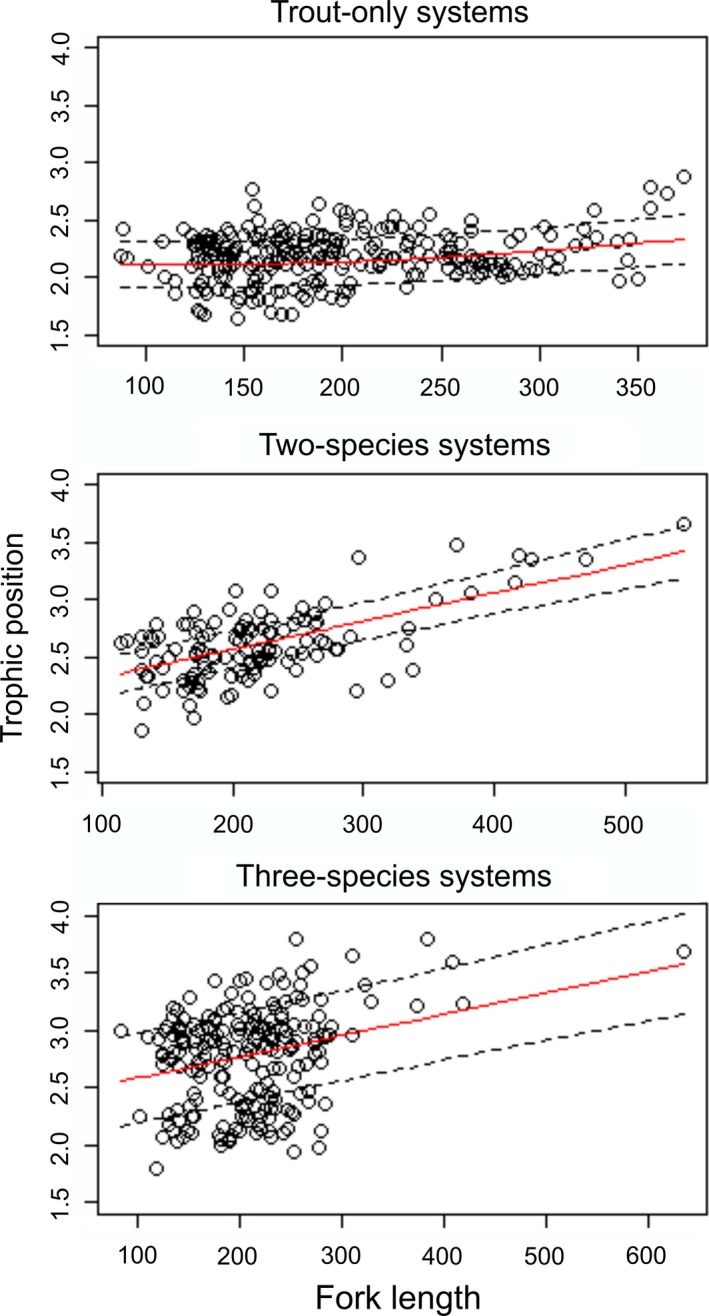
Generalized additive mixed models explaining the variation in trophic position of brown trout over the ontogeny in lakes with different fish communities. Fitted values to the smoothing curve (red line) with 95% confidence bands (broken black line) and observed data (open circles) are shown

No cannibalistic individuals were found in trout‐only systems, whereas piscivory was common in systems with one or both of the other fish species present (Table 1). Relative to the two‐species systems and using pooled data, the prevalence of piscivory was 2.5 times higher in the three‐species systems where sticklebacks were present (7 vs. 18%, respectively). The only distinctive lake‐specific result was found in one three‐species lake (Skilvatn) with an exceptionally high piscivory (see Table 1). Furthermore, the observed minimum size at piscivory did not differ between two‐species (142 mm) and three‐species systems (140 mm). However, the predicted 50% probability shift to piscivory differed between systems, with trout becoming piscivorous at smaller sizes in three‐species than in two‐species systems (390 vs. 540 mm, respectively; Table S4). Piscivory tended to increase with increasing abundance (CPUE) of both stickleback and charr, but no statistically significant relationships were found (*r *=* *.349; *p *=* *.357 and *r *=* *.120; *p *=* *.759, respectively). The trophic position of trout was highest in three‐species systems and lowest in trout‐only systems (Table 1). Overall, the trophic position of trout tended to increase with size regardless of fish community structure (Table 2 and Figure 2).

**Table 1:** Prevalence of piscivory as well as mean ± *SD* estimates of trophic position and individual dietary specialization (1 − PS_i_) of trout among fish community types (trout‐only, two‐species, and three‐species systems)

	Piscivory (prevalence, %)	Trophic position	Individual dietary specialization
Trout only			
Forsanvatn (*n *=* *118)	0.0^a^	2.19 ± 0.16	0.60 ± 0.13
Slunkajavri (*n *=* *54)	0.0^a^	1.90 ± 0.16	0.77 ± 0.11
Storvatn (*n *=* *96)	0.0^a^	2.33 ± 0.14	0.47 ± 0.23
Pooled data (*n *=* *268)	0.0^a^	2.18 ± 0.22	0.58 ± 0.20
Two species			
Fjellfrøsvatn (*n *=* *40)	10.8	2.84 ± 0.32	0.66 ± 0.24
Jernvatnet (*n *=* *56)	4.2	2.48 ± 0.25	0.76 ± 0.16
Sirkelvatn (*n *=* *38)	5.7	2.54 ± 0.24	0.72 ± 0.16
Pooled data (*n *=* *134)	6.7	2.60 ± 0.31	0.72 ± 0.19
Three species			
Makkvatn (*n *=* *78)	16.0	2.31 ± 0.23	0.60 ± 0.16
Skilvatn (*n *=* *47)	46.5	2.97 ± 0.31	0.65 ± 0.15
Takvatn (*n *=* *98)	6.6	3.06 ± 0.21	0.64 ± 0.20
Pooled data (*n *=* *223)	18.2	2.77 ± 0.42	0.62 ± 0.18
Kruskal–Wallis (Pooled data)	–	*Z *=* *239.3; ***p *** **<** *** *** **.001**	*Z *=* *33.6; ***p *** **<** *** *** **.001**

Statistically significant differences (*p *<* *.05) are marked in bold.

Fish prey was not found in trout‐only systems. Data are presented for each lake and community configuration (pooled data).

**Table 2:** Summary of generalized additive mixed models explaining the variation in trophic position (TP) and individual dietary specialization (1 − PS_i_) of trout over the ontogeny, and individual dietary specialization of trout over the TP

	Trout only			Two species			Three species		
	*df*	*F*	*p*	*df*	*F*	*p*	*df*	*F*	*p*
TP over length	1.78	9.71	**<.001**	1.00	94.99	**<.001**	1.00	63.01	**<.001**
1 − PS_i_ over length	1.48	0.43	.42	1.00	0.01	.95	1.00	21.16	**<.001**
1 − PS_i_ over TP	1.00	0.97	.33	1.00	0.22	.64	1.76	6.99	**.001**

Statistically significant differences (*p *<* *.05) are marked in bold.

**Figure 3:**
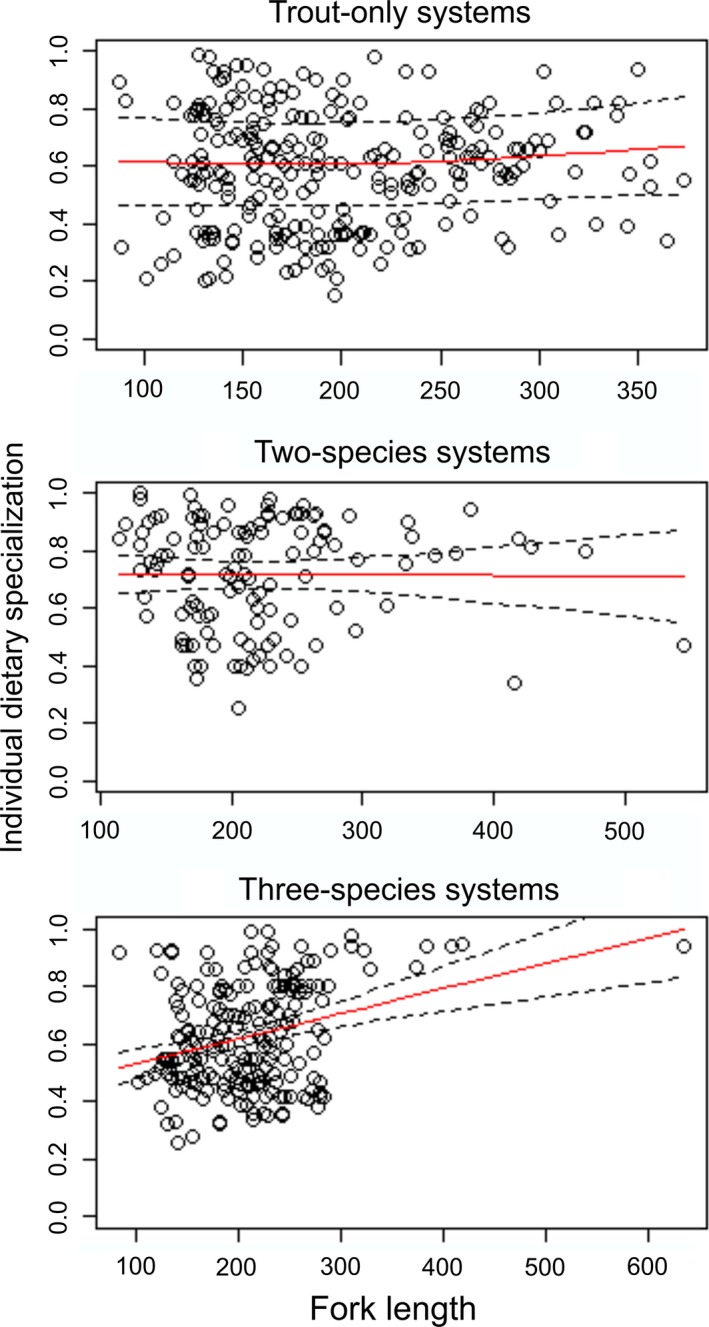
Generalized additive mixed models explaining the variation in individual dietary specialization of brown trout over the ontogeny in lakes with different fish communities. Fitted values to the smoothing curve (red line) with 95% confidence bands (broken black line) and observed data (open circles) are shown

Fish community structure evidently shaped the relationships between individual dietary specialization (1 − PS_i_) and trout size. Individual dietary specialization increased with trout size only in three‐species systems (Table 2 and Figure 3). Despite a slight inverse quadratic effect of size on individual dietary specialization in trout‐only systems, with lowest values at intermediate sizes (Figure 3), the smooth terms were not significant (Table 2). Hence, trout shifted to a more specialized diet during ontogeny only when both mixed competitor–prey (charr) and prey (stickleback) fish species were present.

**Figure 4:**
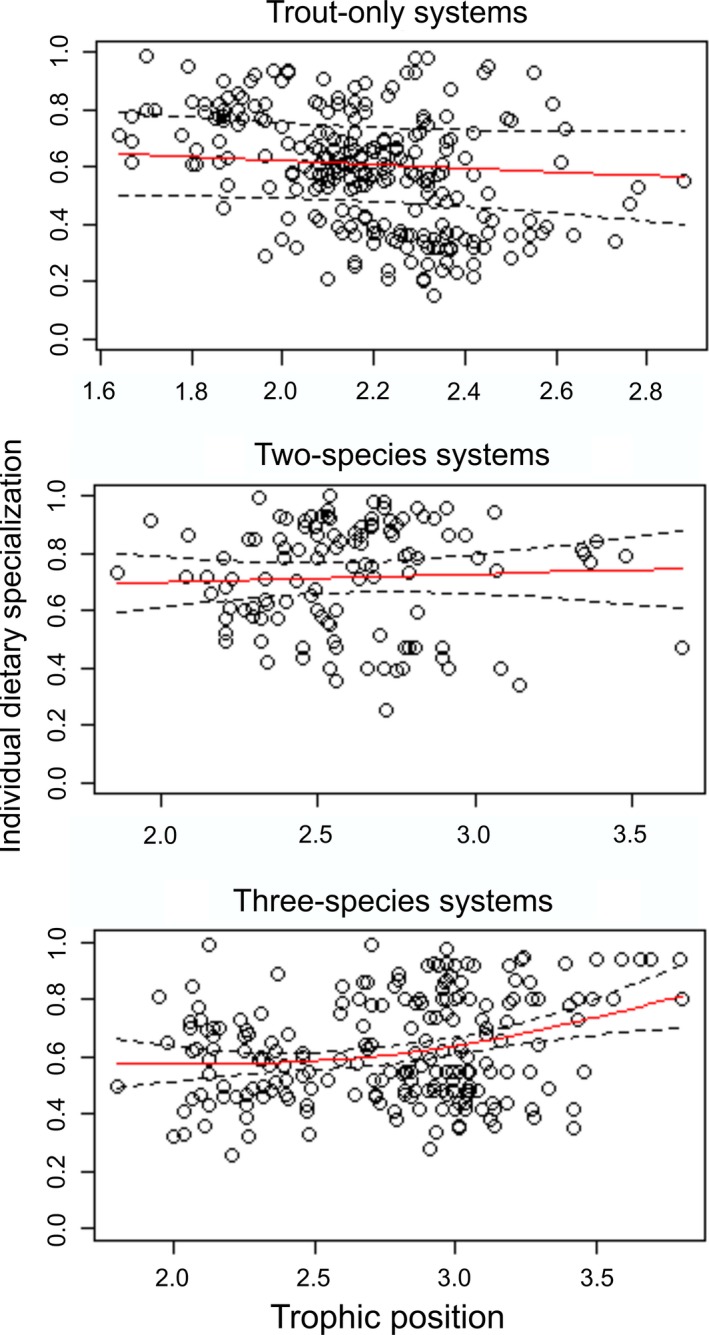
Generalized additive mixed models explaining the variation in individual dietary specialization of brown trout over the trophic position in lakes with different fish communities. Fitted values to the smoothing curve (red line) with 95% confidence bands (broken black line) and observed data (open circle) are shown.

Correspondent to the size relationships, individual dietary specialization increased with trophic position of trout only in the three‐species systems (Table 2). In other words, trout tended to become more specialized with increasing trophic position when a suitable prey fish species (i.e., stickleback) was available (Figure 4). Our sensitivity analyses (excluding individuals of >400 mm fork length found only in two‐ and three‐species systems from the GAMMs) did not alter the results: Trophic position still increased with trout size in both systems, and individual dietary specialization increased with trophic position in three‐species systems (Table S5 and Fig. S3). Moreover, individual dietary specialization increased with trout size in three‐species systems (Table S5), but the smoothness of the curve changed from linear (using pooled data) to inverse quadratic effect after excluding the large individuals (see Figure 3 and Fig. S3 for comparisons).

## 4 Discussion

A central challenge in trophic ecology is to understand the mechanisms that shape trophic ontogeny of species (e.g., Galarowicz et al., 2006; Kimirei et al., 2013). Our study demonstrates that local community structure plays a fundamental role in shaping the ontogenetic dietary shifts in a predatory fish. The presence/absence of a small prey fish species (stickleback) rather than of a mixed competitor–prey fish species (charr) evidently influenced the timing and extent of ontogenetic dietary shifts by trout. Hence, the results suggest that the presence of intermediate consumers (i.e., suitable prey fish for top predators) may facilitate a shift to piscivory, which, in turn, leads to increased dietary specialization and a consecutive increase in trophic position. Intermediate consumers (here stickleback) may thus represent a stepping‐stone for trout in the trophic network, constituting an important intermediate‐sized prey filling the size‐prey gap between invertebrate prey and larger prey (here charr). Overall, the present study gives important insights regarding mechanisms driving ontogenetic niche shifts and variation among individuals within a predatory species.

Our first hypothesis predicting that trout shifts to piscivory particularly in the presence of a profitable prey fish species (sticklebacks) was partly supported. The prevalence of piscivory was higher in the presence of stickleback than in the presence of a mixed competitor–prey fish species (charr), but no marked differences in the observed minimum size at piscivory were found. Nevertheless, our results provide supportive evidence that fish community structure apparently influences piscivorous niche shifts by trout (cf. Eloranta et al., 2015; Jensen et al., 2012; Sánchez‐Hernández & Amundsen, 2015). There has been a growing interest for the period of the ontogeny in which the top predators become piscivorous, particularly because an early transition to piscivory may increase somatic growth and enhance early maturation and lifetime fitness (Jonsson, Naesje, Jonsson, Saksgard, & Sandlund, 1999; Olson, 1996; Post, 2003; Werner, 1986). Piscivory is most common among large trout with a minimum size of 200–300 mm (Grey, 2001; Jensen et al., 2012; Kahilainen & Lehtonen, 2002, 2003), but occurs also at smaller sizes as observed in the present and some previous studies (e.g., Jonsson et al., 1999; L'Abée‐Lund et al., 1992). Although we did not study somatic growth, captured individuals in trout‐only systems were smaller than in two‐species and three‐species systems (maximum lengths of 373, 545, and 634 mm, respectively). This feature may highlight the ecological benefits of individuals acquiring a piscivorous behavior in terms of maximizing the species' growth potential. Individuals which are able to grow large, such as piscivores, may have a positive influence on population dynamics through enhanced recruitment success, because several important maternal traits (e.g., fecundity, egg quality, and egg size) increase with body size (Nicola & Almodóvar, 2002; Venturelli et al., 2010).

Fish community structure appears to influence the trophic position of predatory fish in subarctic lakes, as trophic position of trout increased with increasing number of sympatric fish species (TP_three species_^ ^> TP_two species_ > TP_trout only_). Our study supports the view that the presence of an intermediate consumer (i.e., stickleback) can promote ontogenetic shift to piscivory of top predators and thereby increase food chain length (Eloranta et al., 2015; Post & Takimoto, 2007). In subarctic lakes, piscivory by trout seems to reduce niche overlap and hence resource competition with charr (e.g., Eloranta, Knudsen, & Amundsen, 2013; Sánchez‐Hernández & Amundsen, 2015), but in turn increases direct predation on small charr (Persson et al., 2013). Moreover, we found that the fish community structure shapes individual specialization within the trout populations. While no evident relationships were found in trout‐only or in two‐species systems, individuals tended to become more specialized with increasing size and trophic position in lakes where sticklebacks were present and thus provided suitable prey for piscivorous trout. Although more studies are needed in different ecosystems (cf. Nakazawa, 2015), our study provides novel example of how community structure can affect individual‐level ontogenetic processes.

Our second hypothesis was also supported, because trophic position and individual dietary specialization of trout were correlated only in lakes where sticklebacks were present. Hence, our results demonstrate that community structure shapes the relationship between individual dietary specialization and trophic position. Svanbäck et al. (2015) suggested that high degree of individual diet variation may be connected to increases in the range of trophic positions among individuals. Our findings partially support this view because the trophic position of trout was generally more variable and correlated with individual dietary specialization only in lakes where sticklebacks were present. Although the linkages between individual dietary specialization and trophic position over the species' ontogeny appears to be complex, our study demonstrates that individual‐level ontogenetic processes of top predators can be shaped by the local community structure, in particular by the presence/absence of intermediate consumers (here sticklebacks) rather than of a mixed competitor–prey fish species (here charr). In addition to presence/absence of sympatric species, fish abundance can also be a fundamental factor affecting foraging behavior of top predators when food resources are limited (see, e.g., Sánchez‐Hernández & Cobo, 2013). Hence, in some cases, the ontogenetic niche shifts of top predators might be more affected by the relative abundances of predators, competitors, and prey rather than solely by their presence or absence. This study supports the view that piscivory (prevalence) is positively influenced by increasing abundance of prey fish species (sticklebacks) and mixed competitor–prey fish species (charr). However, this conclusion should be treated with caution as our results were not statistically significant and therefore more studies would be needed to corroborate or refute this conclusion. Additionally, although no information is available about macroinvertebrate abundance or habitat availability in the studied lakes, it seems reasonable to argue that abiotic and biotic environmental factors can have a fundamental importance on the trophic ontogeny of fish species (Olson, 1996; Werner, 1986).

The recent study by Shedd et al. (2015) demonstrates the role of heritable traits in shaping the ontogenetic processes in predatory fishes. In addition to these aspects, our results emphasize the need to acknowledge biotic environmental gradients as community structure evidently modulates the ontogenetic dietary trajectories of predatory trout in subarctic lakes. Hence, our study provides novel insights into the fundamental but complex relationships between trophic position and individual dietary specialization through ontogeny of a predatory fish.

## Conflict of interest

None declared.
