# Supplementary figures and images for: Community structure affects trophic ontogeny in a predatory fish
Source: Ecol Evol. 2016 Dec 20;7(1):358–67. doi: 10.1002/ece3.2600 (PMC5214065; doi:10.1002/ece3.2600)

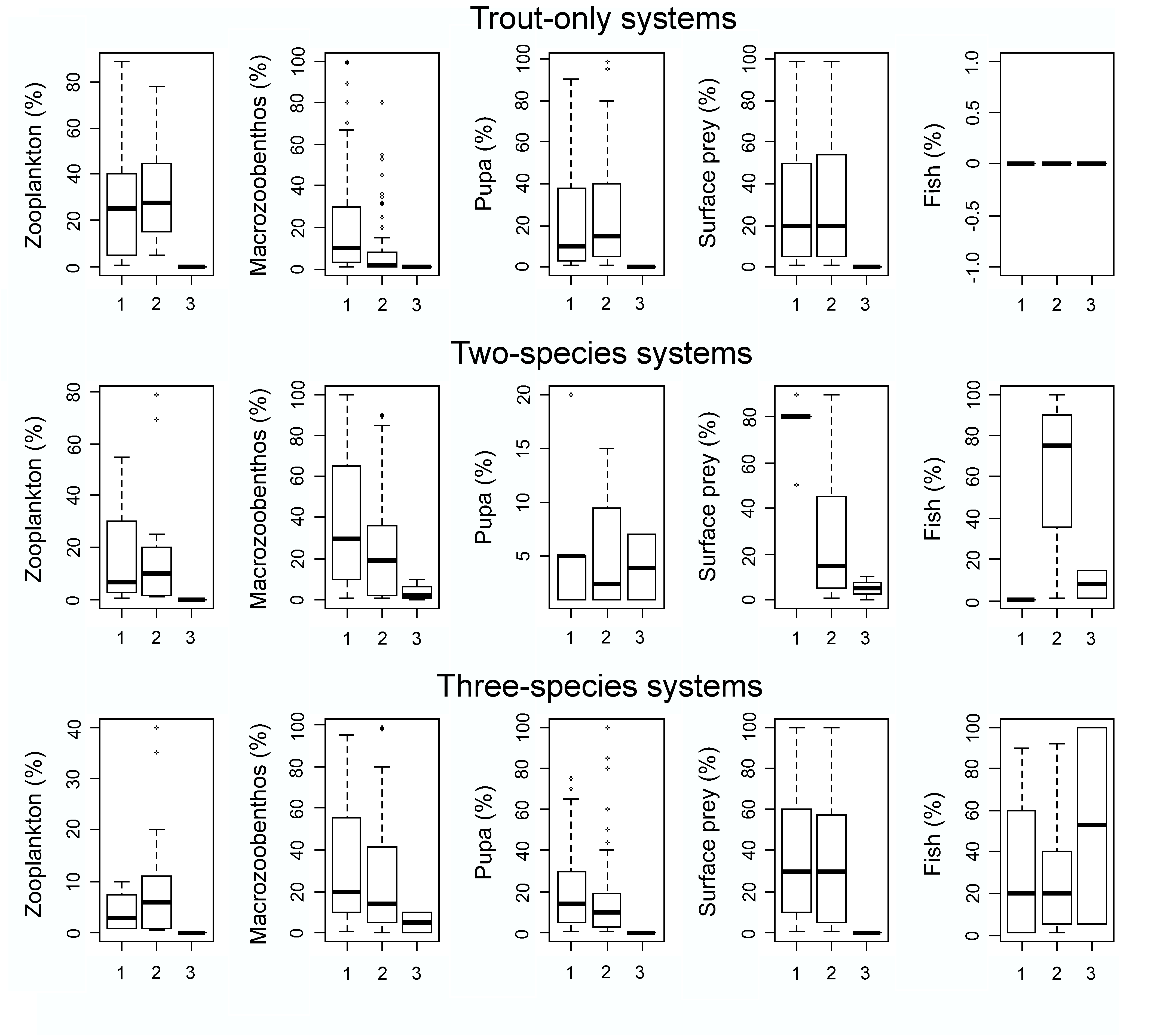

Supplement: Supplementary file 1 [file ECE3-7-358-s001.tif]

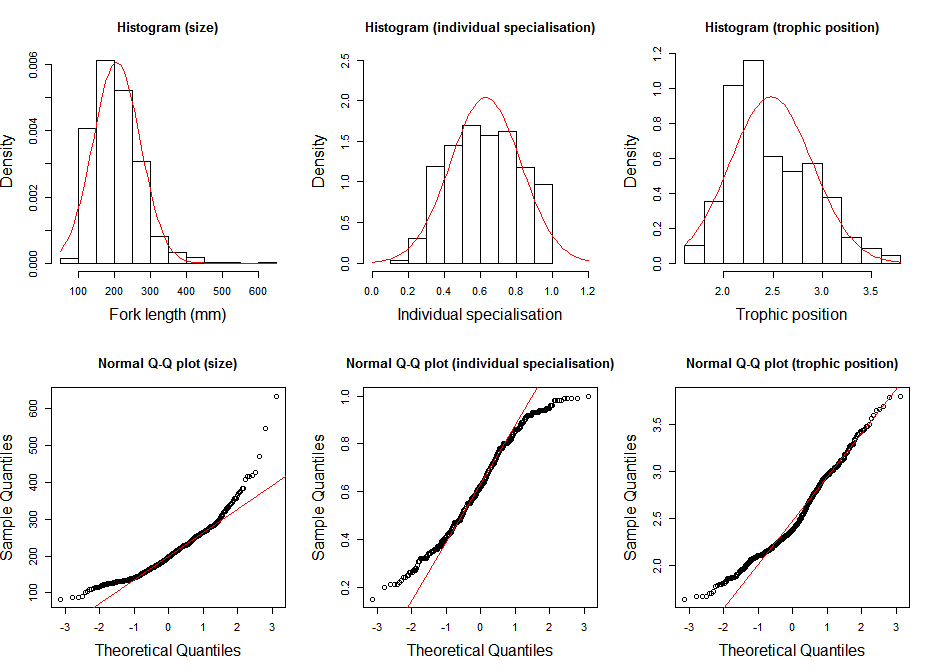

Supplement: Supplementary file 2 [file ECE3-7-358-s002.tiff]

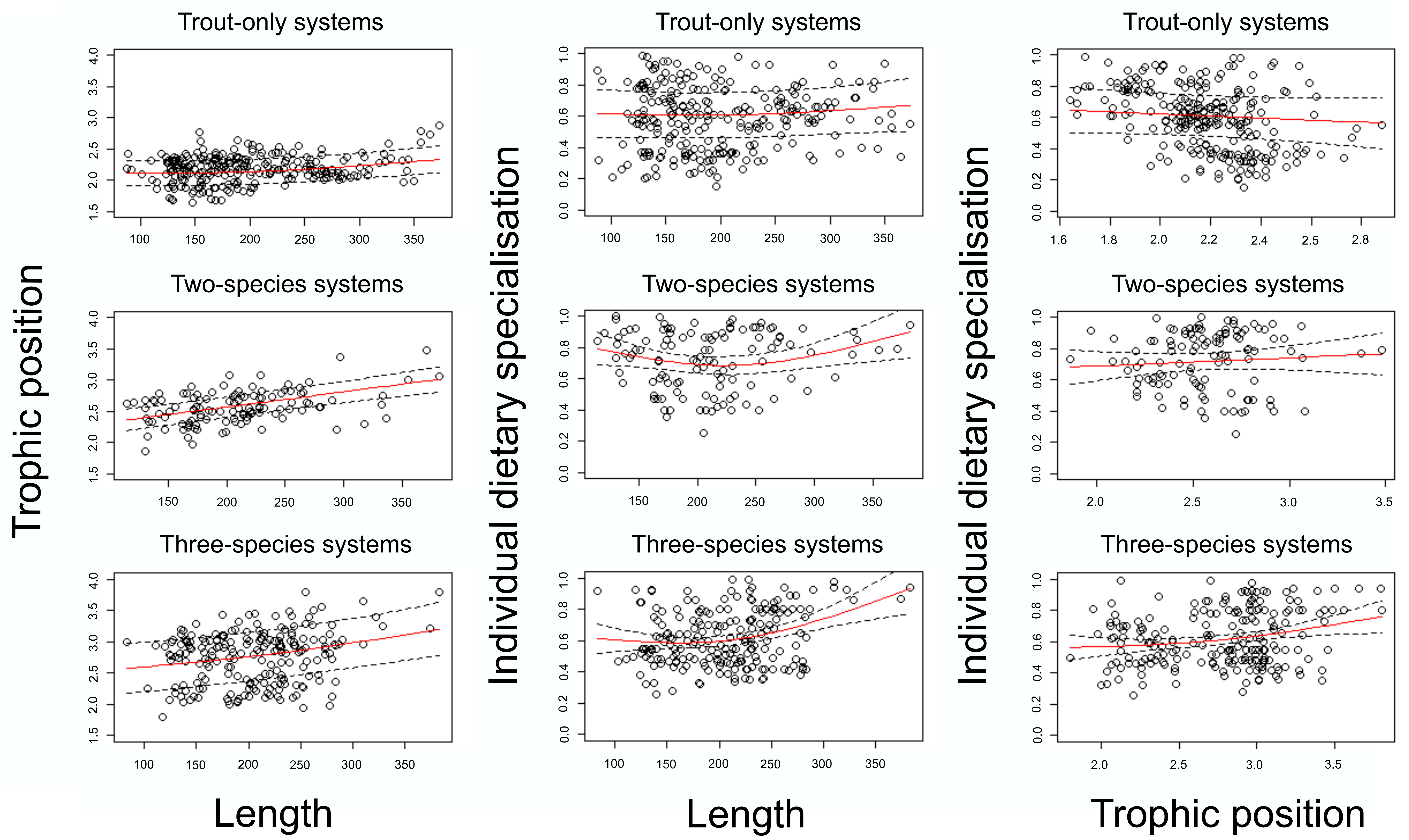

Supplement: Supplementary file 3 [file ECE3-7-358-s003.tif]
