# Supplementary material for: Community structure affects trophic ontogeny in a predatory fish
Source: Ecol Evol. 2016 Dec 20;7(1):358–67. doi: 10.1002/ece3.2600 (PMC5214065; doi:10.1002/ece3.2600)
Supplement: Supplementary file 4 [file ECE3-7-358-s004.docx]

Table S1. Numbers (*n*) and fork lengths (mean with range in parentheses) of trout, charr and stickleback analysed from the nine study lakes with different fish community compositions.

| Lake | Fish community composition | Trout | |  | Charr | |  | Stickleback | |
| --- | --- | --- | --- | --- | --- | --- | --- | --- | --- |
|  |  | *n* | Fork length (mm) |  | *n* | Fork length (mm) |  | *n* | Fork length (mm) |
| Forsanvatn | Trout | 118 | 223 (110–373) |  | – | – |  | – | – |
| Slunkajavri | Trout | 54 | 168 (115–350) |  | – | – |  | – | – |
| Storvatn | Trout | 96 | 179 (87–365) |  | – | – |  | – | – |
| Fjellfrøsvatn | Trout and charr | 40 | 233 (114–545) |  | 226 | 189 (95–415) |  | – | – |
| Jernvatnet | Trout and charr | 56 | 220 (132–382) |  | 72 | 151 (80–307) |  | – | – |
| Sirkelvatn | Trout and charr | 38 | 207 (130–337) |  | 97 | 190 (85–368) |  | – | – |
| Makkvatn | Trout, charr and stickleback | 78 | 208 (103–310) |  | 62 | 216 (93–268) |  | 12 | 40 (20–53) |
| Skilvatn | Trout, charr and stickleback | 47 | 193 (125–270) |  | 127 | 188 (91–328) |  | 36 | 50 (37–59) |
| Takvatn | Trout, charr and stickleback | 98 | 217 (83–634) |  | 146 | 213 (95–443) |  | 51 | 47 (35-60) |
