# Supplementary material for: Community structure affects trophic ontogeny in a predatory fish
Source: Ecol Evol. 2016 Dec 20;7(1):358–67. doi: 10.1002/ece3.2600 (PMC5214065; doi:10.1002/ece3.2600)
Supplement: Supplementary file 5 [file ECE3-7-358-s005.docx]

Table S2. Catch per unit effort (CPUE) of trout, charr and stickleback measured as the number of fish caught per 100 m^2^ of multimesh gillnet per night.

|  | Trout | |  | Trout–charr | | |  | Trout–charr–stickleback | | | |
| --- | --- | --- | --- | --- | --- | --- | --- | --- | --- | --- | --- |
|  | Lake | Trout (CPUE) |  | Lake | Trout (CPUE) | Charr (CPUE) |  | Lake | Trout (CPUE) | Charr (CPUE) | Stickleback (CPUE) |
|  | Forsanvatn |  |  | Fjellfrøsvatn |  |  |  | Makkvatn |  |  |  |
|  | Littoral | 15.5 |  | Littoral | 4.1 | 12.5 |  | Littoral | 24.8 | 3.5 | 2.8 |
|  | Profundal | 0 |  | Profundal | 0.2 | 11.3 |  | Profundal | 0 | 5.0 | 0 |
|  | Pelagic | 2.1 |  | Pelagic | 0 | 2.7 |  | Pelagic | 1.3 | 5.0 | 0 |
|  | Total | 17.6 |  | Total | 4.3 | 26.5 |  | Total | 26.0 | 13.5 | 2.81 |
|  | Slunkajavri |  |  | Jernvatnet |  |  |  | Skilvatn |  |  |  |
|  | Littoral | 2.8 |  | Littoral | 0.7 | 1.2 |  | Littoral | 3.4 | 6.7 | 2.3 |
|  | Profundal | 0 |  | Profundal | 0 | 3.5 |  | Profundal | 0 | 1.3 | 0 |
|  | Pelagic | 0 |  | Pelagic | 0 | 0 |  | Pelagic | 0.2 | 7.7 | 0 |
|  | Total | 2.8 |  | Total | 0.7 | 4.7 |  | Total | 3.6 | 15.6 | 2.3 |
|  | Storvatnet |  |  | Sirkelvatn |  |  |  | Takvatn |  |  |  |
|  | Littoral | 32.8 |  | Littoral | 0.7 | 1.0 |  | Littoral | 6.7 | 5.8 | 5.6 |
|  | Profundal | 0 |  | Profundal | 0 | 1.4 |  | Profundal | 0.2 | 8.1 | 0 |
|  | Pelagic | 0 |  | Pelagic | 0 | 0 |  | Pelagic | 0.1 | 1.3 | 0 |
|  | Total | 32.8 |  | Total | 0.7 | 2.4 |  | Total | 7.0 | 15.2 | 5.6 |
