# Supplementary material for: Community structure affects trophic ontogeny in a predatory fish
Source: Ecol Evol. 2016 Dec 20;7(1):358–67. doi: 10.1002/ece3.2600 (PMC5214065; doi:10.1002/ece3.2600)
Supplement: Supplementary file 6 [file ECE3-7-358-s006.docx]

Table S3. Results from Shapiro–Wilk tests indicating non-normal distribution of the data (*P*< 0.05 in most cases).

|  | Size (fork length) | Individual specialisation | Trophic position |
| --- | --- | --- | --- |
| Trout-only | *W* = 0.941; ***P* < 0.001** | *W* = 0.975; ***P* < 0.001** | *W* = 0.991; *P* = 0.111 |
| Two-species | *W* = 0.872; ***P* < 0.001** | *W* = 0.933;***P* < 0.001** | *W* = 0.964; ***P* = 0.003** |
| Three-species | *W* = 0.886; ***P* < 0.001** | *W* = 0.961;***P* < 0.001** | *W* = 0.968; ***P* < 0.001** |
| Pooled data | *W* = 0.920; ***P* < 0.001** | *W* = 0.976; ***P* < 0.001** | *W* = 0.965; ***P* < 0.001** |
