# Supplementary material for: Community structure affects trophic ontogeny in a predatory fish
Source: Ecol Evol. 2016 Dec 20;7(1):358–67. doi: 10.1002/ece3.2600 (PMC5214065; doi:10.1002/ece3.2600)
Supplement: Supplementary file 7 [file ECE3-7-358-s007.docx]

Table S4. Summary of logistic regression models explaining the probability of piscivorous behaviour over trout’s ontogeny for the two-species and three-species systems (no piscivory was observed in trout-only systems). Statistically significant differences (*P*< 0.05) are marked in bold.

|  | Model parameters | | | |  |  | Probability of piscivory (mm) | | |
| --- | --- | --- | --- | --- | --- | --- | --- | --- | --- |
|  | Variable | Coefficient | S.E. | *P* value |  |  | 25% | 50% | 75% |
| Two-species | Intercept | -4.842 | 1.081 | **<0.001** |  |  | 420 | 540 | 665 |
|  | Fork length | 0.009 | 0.004 | **0.016** |  |  |  |  |  |
| Three-species | Intercept | -3.453 | 0.697 | **<0.001** |  |  | 260 | 390 | 510 |
|  | Fork length | 0.009 | 0.003 | **0.003** |  |  |  |  |  |
