# Supplementary material for: Community structure affects trophic ontogeny in a predatory fish
Source: Ecol Evol. 2016 Dec 20;7(1):358–67. doi: 10.1002/ece3.2600 (PMC5214065; doi:10.1002/ece3.2600)
Supplement: Supplementary file 8 [file ECE3-7-358-s008.docx]

Table S5. Summary of generalized additive mixed models (*GAMMs*) explaining the variation in trophic position (*TP*) and individual dietary specialisation (1–*PS_i_*) of trout over the ontogeny, and individual dietary specialisation of trout over the trophic position after excluding individuals of >400 mm fork length. Statistically significant differences (*P*< 0.05) are marked in bold. No individuals of >400 mm fork length were found in trout-only systems (see Table 2 for model parameters of trout-only systems).

|  |  | Two-species | | |  | Three-species | | |
| --- | --- | --- | --- | --- | --- | --- | --- | --- |
|  |  | d.f. | *F* | *P* value |  | d.f. | *F* | *P* value |
| *TP* over length |  | 1.00 | 47.17 | **<0.001** |  | 1.49 | 29.38 | **<0.001** |
| 1–*PS_i_* over length |  | 1.86 | 2.77 | 0.055 |  | 1.85 | 7.31 | **<0.001** |
| 1–*PS_i_* over *TP* |  | 1.00 | 0.59 | 0.445 |  | 1.53 | 5.22 | **0.007** |
